# Renal Colic: A Red Herring for Mucocele of the Appendiceal Stump

**DOI:** 10.1155/2018/2502183

**Published:** 2018-12-06

**Authors:** Sameera Ganti, Pothiawala Sohil

**Affiliations:** ^1^SingHealth Emergency Medicine Residency Program, Singapore; ^2^Department of Emergency Medicine, Singapore General Hospital, Singapore

## Abstract

Flank pain with hematuria is a common presentation in the emergency department. The commonest differential diagnosis of these patients is renal/ureteric calculus or pyelonephritis. These patients are usually treated with analgesia, antibiotics in case of pyelonephritis, and are discharged with an outpatient referral to a urologist. This case report describes a 51 year old male who presented to the ED for recurrent flank pain and hematuria. Bedside ultrasonography in the ED demonstrated a cystic lesion in the renal area. CT urography revealed an appendiceal stump mucocele and patient was transferred under surgical care. This case highlights the importance of the utility of bedside ultrasound in patients presenting to the ED with flank pain or abdominal pain which can lead to expedited assessment and appropriate management.

## 1. Introduction

Flank pain is a common presentation in the emergency department (ED). This symptom coupled with a history of hematuria raises the suspicion of a renal/ureteric calculus among most clinicians. Some patients who have an associated complaint of dysuria with or without fever are diagnosed to have pyelonephritis. However, there are many conditions that mimic this presentation. We present the case of a middle-aged patient who presented with flank pain with suspicion of renal colic.

## 2. Case Report

A 51 year old man presented to the ED for the third time in 2 weeks with complaints of flank pain and hematuria. He had complained of left sided flank pain during the initial 2 visits. During the first visit, the patient was diagnosed to have renal colic. X-ray KUB did not show any renal stone. He was treated symptomatically with analgesia and discharged. During the second visit with complaints of persistent left flank pain despite taking analgesia given at discharge, he was admitted to the emergency observation ward for pain management. Bedside ultrasound done then was noted to have mild left sided hydronephrosis. He was pain free at the end of the observation and was then discharged with analgesia and an outpatient follow-up with the urology department. He was also scheduled to have an outpatient computed tomography scan of the kidneys, ureters, and bladder (CT KUB). However, 2 days before the scheduled CT, he represented to the ED with right sided flank pain since morning on the day of his visit. The pain radiated to the right groin and was associated with hematuria. He was not passing blood clots. He denied any other complaints of fever, weight loss, vomiting, diarrhea, or constipation. He had a past medical history of hypertension, diabetes mellitus, and hyperlipidemia. He had previous surgeries for appendicectomy and cholecystectomy.

His vital signs were stable. There was tenderness over the right flank on physical examination. There was also a palpable tender mass measuring about 5x5cm over the right lumbar region. There was no renal angel tenderness. Bedside ultrasound in the ED showed an appearance of a 6 cm cystic lesion around the inferior pole of the right kidney with internal echogenicity within the cyst. The provisional diagnosis was a bleeding renal cyst versus a tumor. Blood tests done in the ED showed a mildly elevated white cell count 12.64 × 10(9), hemoglobin of 14 gm/dL, and creatinine of 109 Umol/L.

Patient was seen by the urology on-call in the ED and his bedside ultrasound done by the urologist revealed a 10 cms cystic lesion anterior to the lower pole of the right kidney with irregular internal echogenicity. Patient was admitted to urology department for further evaluation and management.

**Figure 1:**
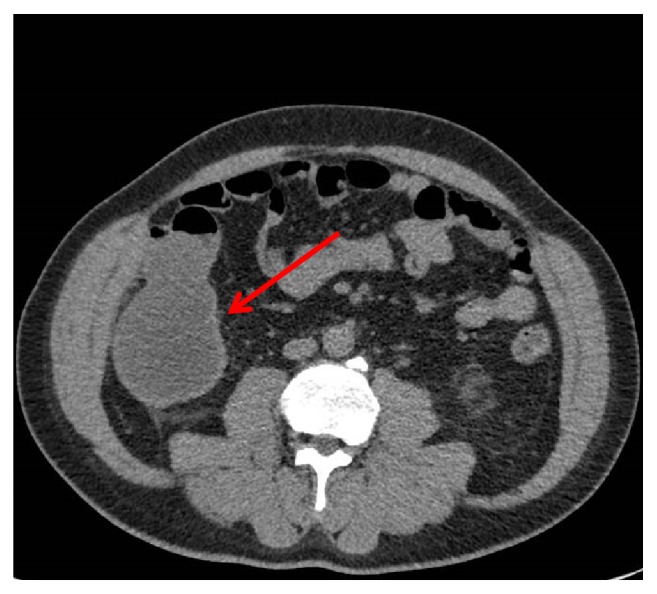
CT KUB showing the cystic swelling arising from the appendiceal stump (red arrow).

Inpatient CT Intravenous Pyelography (IVP) was done and the left kidney showed relative hypoenhancement and reduced excretion of contrast, associated with diffuse ureteric thickening and periureteric fat stranding which could be related to a passed left renal calculus or a differential diagnosis of ureteropyelonephritis which was less likely due to diffuse involvement of left kidney. There was a tubular cystic structure in the right flank with inferior tip at the same site of previously inflamed appendix stump base which could represent mucocele of the appendix stump (Figure 1).

**Figure 2:**
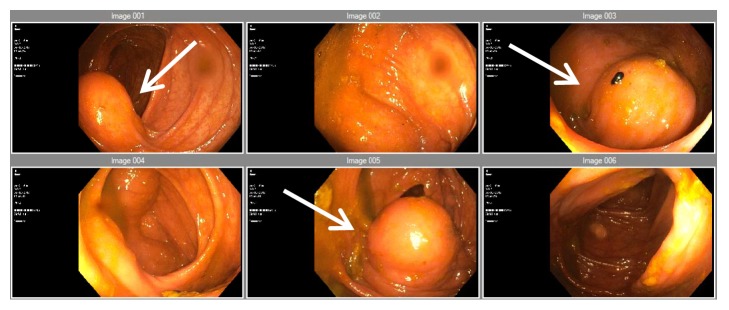
Extraluminal compression caused by the mucocele seen during the colonoscopy (white arrows).

Patient was then transferred under the care of the general surgery department, where he underwent a colonoscopy which demonstrated an extrinsic compression at the caecum and appendiceal orifice and a smooth pedunculated polyp measuring 10 mm in the sigmoid colon (Figure 2). Exploratory laparotomy was done, the mucocele was resected, and patient recovered well. Histopathology revealed a benign mucinous cystadenoma. The patient was discharged well with an outpatient surgical follow-up.

## 3. Discussion

An appendiceal mucocele is a well-known but rare entity, where the appendiceal lumen is distended with mucinous material. The incidence of this is 0.2-0.3% of all appendiceal specimens [1]. Up to 50% of patients with an appendiceal mucocele may be asymptomatic [2]. Symptoms range from right iliac fossa pain to mass per abdomen [3]. The more symptomatic a patient, the higher the likelihood of malignancy [4] and this is why the diagnosis and the subsequent management of this condition are so important.

Appendiceal stump mucoceles are an even rarer entity, with only 4 previous reported cases in literature. Yeong et al. were the first to report a case of an appendiceal stump mucocele causing pseudomyxoma peritonei [5]. The reports by Lien et al., Korkolis et al., and El Ajmi et al. are of a benign cystadenoma of the appendiceal stump where the patient presented with symptom of right lower quadrant pain [6–8]. Our case is the first of its kind where the patient presents with flank pain and hematuria which would be most commonly diagnosed as renal or ureteric colic.

Management of both conditions, namely, appendiceal mucocele and appendiceal stump mucocele, is aimed at surgical resection and histopathological guided therapy. In retrospective study, there was an association found between appendiceal mucoceles and colonic neoplasms and that study recommends surveillance colonoscopy in patient diagnosed with appendiceal mucoceles [4]. This diagnostic strategy was applied to our patient who underwent a colonoscopy which did not reveal any pathology.

The four types of pathological findings described in appendiceal mucoceles are (1) mucous hyperplasia, (2) simple mucocele, (3) cystadenoma, and (4) cystadenocarcinoma [9]. The phenomenon of pseudomyxoma peritonei is well associated with the presence of an appendiceal/appendiceal stump mucocele [10]. This is a dreaded complication and to avoid it, prompt surgical management is indicated.

Emergency ultrasonography (EUS) has become a standard of care in the management of patients in the emergency department. Guidelines published by the American College of Emergency Physicians encourage the use of EUS for therapeutic, resuscitative, and diagnostic purposes [11]. Bedside ultrasonography is a quick and easy method to assess patients with suspected nephrolithiasis and is associated with diagnostic accuracy and less radiation exposure [12]. Marzec et al. and Garcia et al. describe a case of renal tumor detected in the ED by using EUS for assessment of suspected renal colic [13, 14]. They suggested that EUS is cheaper and faster and a good screening tool, prior to definitive imaging such as a CT scan.

## 4. Conclusion

This case highlights the importance of the utility of bedside ultrasound in patients presenting to the ED with flank pain or abdominal pain. It is a quick, focused and an invaluable tool and has become the standard of care in the EDs and also is part of the training for emergency medicine residents in the assessment of patients presenting with abdominal pain to the ED. Further evaluation prompted by bedside EUS done in the ED can lead to an expedited assessment and appropriate patient management, thus improving patient outcomes.
